# C-756-07. Twice as Long, Half the Risk: PICCs Outperform CVCs in Pediatric Burn Patients

**DOI:** 10.1093/jbcr/irag033.051

**Published:** 2026-04-06

**Authors:** Christian Hudson-Bradford, Samantha Carranza, Megan Zacarias, Elika Ridelman, Justin D Klein, Christina M Shanti

**Affiliations:** Children's Hospital - Michigan, Detroit, MI; Wayne State University School of Medicine, Detroit, MI; Wayne State University School of Medicine, Detroit, MI; Wayne State University School of Medicine, Children's Hospital of Michigan Burn Center, Detroit, MI; Wayne State University School of Medicine, Children's Hospital of Michigan Burn Center, Detroit, MI; Wayne State University School of Medicine, Children's Hospital of Michigan Burn Center, Detroit, MI

## Abstract

**Introduction:**

Venous access is critical in burn care for fluid resuscitation, medication delivery, and nutritional support. While central venous catheters (CVCs) are traditionally used, peripherally inserted central catheters (PICCs) offer an alternative option with a potentially favorable safety profile, longer dwell time, and reduced need for re-insertion. This study compares patient characteristics, complication rates, and catheter duration between PICC and CVC lines in pediatric burn patients, advocating for early PICC placement when feasible.

**Methods:**

We performed a retrospective review of 140 pediatric burn patients with central venous access from 2015-2024, categorizing them into PICC and CVC cohorts. Variables collected included demographics, burn characteristics (Total Body Surface Area (TBSA) for 2nd/3rd-degree burns), hospital length of stay (LOS), ICU days, catheter dwell time (days from insertion to removal), and line-related complications (expressed per 1000 line-days). Catheter duration was compared with Welch’s t-test and Mann–Whitney U test; effect size was reported as Cohen’s d. Duration data was available for 106 PICCs and 120 CVCs; complication counts were derived from 107 PICCs and 121 CVCs.

**Results:**

The cohort contributed 2095.6 PICC line-days and 1160.4 CVC line-days. Complication rates per 1000 line-days were generally lower for PICCs than CVCs: total complications (8.6 vs 23), CLABSI (5.25 vs 16.37), thrombosis (0.95 vs 1.72), and dislodgement (0.95 vs 0.86). Malfunction and pneumothorax occurred only in CVCs (1.72 each), while site infections were slightly higher in PICCs (1.43 vs 0.86).

PICC dwell time was much longer than CVCs: mean 19.8 ± 19.2 vs 9.2 ± 6.6 days (median 14.5 vs 8; IQR 7.25–25 vs 5.5–12; range 0–99 vs 0–42). The difference was significant (Welch t = –5.38, *p*=3.5 × 10^-7^; Mann–Whitney U = 3872, *p*=5.8 × 10^-7^; Cohen’s d = 0.73).

Patients requiring CVCs had higher mean TBSA (32.6 % vs 25.5 %), lower mean BMI (16.2 vs 19.7 kg/m^2^), and were more often intubated/ventilated (69.2 % vs 55.1 %).

**Conclusions:**

PICC lines demonstrated a markedly longer dwell time (≈ 20 days vs 9 days) and a more favorable complication profile, with significantly lower rates of total complications and CLABSI. Although PICCs showed slightly higher dislodgement and site-infection rates, the reduction in severe complications and the need for fewer re-insertions outweigh these minor drawbacks. Higher TBSA and ventilator use in the CVC cohort underscore that sicker patients may still require initial CVC placement, yet transitioning to a PICC once large-bore access is no longer critical offers both safety and efficiency advantages.

**Applicability of Research to Practice:**

PICC lines provide a superior safety profile and nearly double the dwell time relative to CVCs in pediatric burn patients. We recommend converting CVCs to PICCs as soon as clinically feasible to mitigate complication risk, decrease catheter turnover, and improve overall outcomes.

**Funding for the study:**

Foundation Funding.

